# Pu-erh tea attenuates obesity by remodeling gut microbiota and activating energy expenditure

**DOI:** 10.3389/fmicb.2025.1752456

**Published:** 2026-01-15

**Authors:** Xiujuan Jiang, Fan Wu, Mianhong Xu, Liyong Luo, Liang Zeng

**Affiliations:** Integrative Science Center of Germplasm Creation in Western China (CHONGQING) Science City, College of Food Science, Southwest University, Chongqing, China

**Keywords:** energy expenditure, energy intake, gut microbiota, obesity, tea

## Abstract

**Introduction:**

Energy excess-induced obesity has emerged as a global public health concern, and Pu-erh tea has attracted extensive research interest due to its prominent anti-obesity potential. This study aimed to investigate the anti-obesity activity of Pu-erh tea extract (PTE) and its underlying molecular mechanisms.

**Methods:**

High-fat diet (HFD)-fed mice were used as the experimental model and subjected to Pu-erh tea extract (PTE) intervention. Whole-body energy metabolism of the mice was monitored using metabolic cages; morphological analysis was performed to characterize the lipid droplet distribution in brown adipose tissue (BAT); transcriptomic and lipidomic techniques were integrated to systematically detect changes in the gene expression profile and lipid composition in BAT; and 16S rRNA gene sequencing was employed to determine alterations in the gut microbiota.

**Results:**

PTE intervention significantly ameliorated HFD-induced obesity in mice without affecting food intake. Metabolic cage monitoring results demonstrated that PTE increased whole-body energy expenditure in mice and shifted the preference of energy substrate utilization toward lipid metabolism. Morphological observation of BAT revealed that lipid droplets in BAT of PTE-treated mice were mainly concentrated in the small-diameter range, which is consistent with the phenotypic characteristic of activated BAT function. Transcriptomic and lipidomic analyses indicated that PTE significantly activated mitochondrial fatty acid *β*-oxidation and respiratory electron transport in BAT, while markedly reducing the relative contents of lipids such as triglycerides (TG), diglycerides (DG), phosphatidylethanolamine (PE), and phosphatidylcholine (PC). Additionally, 16S rRNA gene sequencing analysis showed that PTE significantly decreased the Firmicutes/Bacteroidetes (F/B) ratio, enhanced the abundance of probiotics, and decreased the abundance of harmful bacteria.

**Conclusion:**

Under HFD conditions, the anti-obesity effect of PTE is dependent on the synergistic effect of BAT metabolic remodeling and gut microbiota regulation. PTE activates BAT thermogenic function, promotes fatty acid oxidation, increases gut microbiota richness, and reduces the F/B ratio. These effects collectively enhance systemic energy metabolism and ultimately ameliorate obesity phenotypes. This study provides critical theoretical support for the development of PTE-based anti-obesity functional preparations and lays a foundation for subsequent in-depth exploration of specific molecular targets through which PTE regulates BAT function.

## 1 Introduction

Obesity has emerged as a major global public health challenge, and its prevalence continues to escalate. The World Obesity Atlas 2023 projects that by 2035, approximately 51% of the global population (i.e., 4.005 billion people) will be overweight or obese (Lobstein et al., 2023). The core pathology of obesity lies in energy excess caused by impaired energy metabolism (Gao F. et al., 2024; Goldfine and Shoelson, 2017). Brown adipose tissue (BAT), with its unique energy-consuming function (Bornstein et al., 2023), has become a key potential target for improving obesity.

Unlike white adipose tissue (WAT) that is primarily responsible for energy storage, BAT serves as the core thermogenic organ of the body. Its key mechanism of action is to maintain and restore whole-body energy metabolic homeostasis through non-shivering thermogenesis (Bornstein et al., 2023). Among the thermogenic pathways of BAT, the uncoupling protein 1 (UCP1)-dependent pathway is the primary mechanism (Johnson et al., 2023). UCP1 is an integral membrane protein primarily localized to the inner mitochondrial membrane of BAT. It decouples nutrient oxidation from ATP synthesis, leading to the direct dissipation of energy as heat (Rosina et al., 2022; Whitehead et al., 2021). Beyond this classical pathway, multiple UCP1-independent thermogenic mechanisms exist in BAT (Cicuéndez et al., 2025; Lee et al., 2025), including peroxisomal thermogenesis (Liu et al., 2025), calcium cycling-mediated thermogenesis (Auger et al., 2025), and futile creatine cycling-induced thermogenesis (Rahbani et al., 2021). These pathways collectively form the thermogenic regulatory network of BAT. Notably, BAT whitening is closely associated with metabolic dysfunction (Wang G. et al., 2025; Zammit et al., 2025). Accumulating evidence has confirmed that BAT whitening is often accompanied by reduced oxidative phosphorylation efficiency, disrupted fatty acid metabolism, and mitochondrial dysfunction (Zammit et al., 2025). Thus, improvements in BAT morphology may also indicate the normalization of its metabolic function.

Pu-erh tea, a traditional post-fermented dark tea from *Camellia sinensis*, is rich in polyphenols (Zhang et al., 2012) and caffeine (Lin et al., 2021) and has anti-obesity (Ning et al., 2025), antioxidant (Zhang et al., 2012), and hypolipidemic activities (Qin et al., 2022; Tung et al., 2022). Existing studies have demonstrated that bioactive components in Pu-erh tea, such as tea polyphenols, theabrownins and tea polysaccharides, exert anti-obesity effects through multiple pathways. These include reducing blood lipid levels (Zhang et al., 2024), regulating gut microbiota and their metabolites (Li et al., 2025; Shan et al., 2025), alleviating systemic inflammation (Li et al., 2016; Wang et al., 2020), activating BAT thermogenesis (Wang et al., 2021), and promoting white adipose tissue browning and fatty acid *β*-oxidation (Liao et al., 2022; Rocha et al., 2016; Wang et al., 2012). However, previous studies have failed to link the activation of BAT thermogenesis by Pu-erh tea to systemic metabolic status.

Therefore, the present study employed a high-fat diet-induced obese mouse model and systematically elucidated the anti-obesity mechanisms of Pu-erh tea extract (PTE) through systematic analysis of whole-body lipid levels, metabolic cage monitoring, BAT morphological, transcriptomic, and lipidomic analyses, as well as 16S rRNA gene sequencing. Our results demonstrated that Pu-erh tea significantly remodels the gut microbiota, enhances lipid mobilization and utilization, and alters energy utilization substrates, thereby increasing energy expenditure and inhibiting systemic lipid accumulation. In summary, this study has more comprehensively delineated the molecular mechanisms underlying the anti-obesity effects of Pu-erh tea and provides a theoretical foundation for the future development of nutritional interventions targeting the microbiota and BAT.

## 2 Materials and methods

### 2.1 Materials and reagents

Glacial acetic acid, acetonitrile, sodium acetate, tetrahydrofuran, and methanol were high performance liquid chromatography (HPLC) grade. The standards of amino acids, catechins, gallic acid, and caffeine were guaranteed reagents. All the above reagents were purchased from Sigma-Aldrich, Inc. (St. Louis, MO, USA). The reagents used in the UHPLC-QTOF-MS experiments, including methanol, acetonitrile, formic acid, water, and isopropanol were purchased from Fisher Chemical (Pittsburgh, PA, USA). Commercial kits, which include total triglyceride (TG), total cholesterol (TC), low-density lipoprotein cholesterol (LDL-C), high-density lipoprotein cholesterol (HDL-C) and leptin were purchased from Nanjing Jiancheng Bioengineering Institute (Nanjing, Jiangsu, China).

### 2.2 Preparation of PTE and analysis of its main components

Pu-erh tea (7,572 ripened tea) was purchased from Yunnan TEATEA Group, and the production year was 2006. This ripe Pu-erh tea was selected instead of raw Pu-erh tea primarily due to its unique pile fermentation process and the derived bioactive component characteristics. The pile fermentation exclusive to ripe Pu-erh tea promotes the formation of theabrownin, a key bioactive component that has been confirmed to regulate lipid metabolism by modulating gut microbiota and microbiota-derived metabolites (Shan et al., 2025; Xiao et al., 2024). Additionally, the post-fermentation process stabilizes the chemical composition of ripe Pu-erh tea, minimizing batch-to-batch variations of bioactive components and ensuring experimental specificity and reproducibility, which is crucial for this study focusing on lipid metabolism regulation.

The sample was extracted with ultrapure water at a ratio of 1:15 (w/v) for 45 min in boiling water. After filtration, ultrapure water (1:10 (w/v)) was added to the filter residue for extraction for 30 min. The filtrate was combined and then freeze-dried to obtain PTE, which was subsequently stored at −80 °C (Hu et al., 2022).

The main biochemical components of PTE were determined via high-performance liquid chromatography and biochemical methods (Hu et al., 2022). The contents of PTE are shown in Supplementary Table S1.

### 2.3 Animals and experimental design

Twenty-four 6-week-old male specific pathogen-free C57BL/6 mice (weighing 17 ± 2 g) were purchased from the experimental animal center at Chongqing Medical University. The animals were maintained with a 12 h daylight cycle and provided autoclaved food (Jiangsu Xietong Pharmaceutical Bioengineering Co., Ltd.) and distilled water or PTE (0.25%, w/v) ad libitum (Hu et al., 2022). After 1 week of acclimatization, the mice were randomly assigned to four groups (*n* = 6 per group), i.e., a chow diet (Chow), a high-fat diet (HFD), a chow diet supplemented with PTE (Chow_T) and a high-fat diet supplemented with PTE (HFD-T). The composition of the feeds is shown in Supplementary Table S2. The mice in the Chow_W and HFD_W groups drank water freely. The mice in the Chow_T and HFD_T groups drank 0.25% (w/v) PTE freely. The air conditions were controlled at a temperature of 23 ± 2 °C and 55–65% relative humidity. All the animal studies were approved by the Experimental Animal Ethical Review Committee of Southwest University (approval no. IACUC-20231228-06). After 16 weeks, metabolic cage monitoring was performed. Following an overnight fast, the mice were sacrificed via CO₂ inhalation anesthesia, and serum, intestinal contents, and adipose tissue were collected for subsequent analyses. Serum was stored at −20 °C, whereas intestinal contents and adipose tissue were stored at −80 °C.

### 2.4 Indirect calorimetry

Oxygen consumption (VO₂), carbon dioxide production (VCO₂), heat generation, food intake, water intake and locomotor distance were continuously monitored in mice (*n* = 4 per group) via the Comprehensive Lab Animal Monitoring System (CLAMS) (Duregon et al., 2023). Prior to data collection, the mice were acclimated in groups for 24 h. The animals were subsequently weighed, assessed for general health status, and individually housed in metabolic chambers for continuous recording of metabolic parameters.

### 2.5 Serum biochemical analysis

Whole blood was centrifuged at 4 °C and 3,000 r/min for 15 min, and the supernatant was then carefully collected (Hu et al., 2025). Blood lipid levels and leptin levels were measured using the corresponding assay kits.

### 2.6 Hematoxylin and eosin (H&E) staining

BAT samples fixed in 4% paraformaldehyde were dehydrated through graded ethanol, cleared in xylene, embedded in paraffin, and sectioned at 4 μm. The sections were deparaffinized, rehydrated, stained with Harris hematoxylin (5 min), differentiated with 1% acid alcohol (3 s), rinsed, counterstained with 1% eosin (1 min), dehydrated, cleared, and mounted with neutral resin (Hu et al., 2022). Histological images were acquired via a light microscope (Olympus, Japan), and the adipocyte area and lipid droplet diameter were quantified via ImageJ.

### 2.7 Lipidomic analysis of BAT

*Lipid extraction* (Gao R. et al., 2024): Approximately 50 mg of adipose tissue was homogenized in 280 μL of methanol:water (2:5, v/v) and 400 μL of MTBE with a stainless-steel bead using a tissue grinder (Wonbio-96c, 50 Hz, −10 °C, 6 min), followed by sonication (40 kHz, 30 min, 5 °C). After incubation at −20 °C (30 min) and centrifugation (13,000 × g, 15 min, 4 °C), the upper phase (∼350 μL) was collected, dried under nitrogen, and reconstituted in 100 μL of isopropanol:acetonitrile (1:1, v/v). The supernatant was centrifuged and transferred to vials for UHPLC–MS/MS analysis (2 μL injection).

*Quality control*: A pooled QC sample, prepared by mixing equal volumes of all samples, was injected every six samples to monitor instrument stability.

UHPLC–MS/MS analysis: Lipidomics was performed on a Thermo Vanquish UHPLC coupled to a Q Exactive HF–X Orbitrap using an Accucore C30 column (100 × 2.1 mm, 2.6 μm). Mobile phases: A, 10 mM ammonium acetate in acetonitrile:water (1:1) with 0.1% formic acid. B, 2 mM ammonium acetate in acetonitrile:isopropanol:water (10:88:2) with 0.02% formic acid. A 20-min gradient was applied at 0.4 mL/min and 40 °C. MS was operated in both positive and negative ion modes with stepped NCE (20/40/60) and DDA acquisition over 200–2000 m/z.

*Data preprocessing and annotation*: The data were analyzed through the free online platform of the majorbio cloud platform^1^ (Ren et al., 2022). The raw data were processed via LipidSearch with 10 ppm mass tolerance. Features present in ≥80% of the samples were retained. Missing values were imputed, and intensities were sum-normalized. Variables with an RSD > 30% in the QC were excluded. The data were log10-transformed.

Differential lipids were identified via PCA, OPLS-DA (*ropls* package), Student’s *t* test, and the VIP score (>1, *p* < 0.05). KEGG pathway enrichment was performed via Fisher’s exact test,^2^ highlighting pathways associated with metabolic regulation.

### 2.8 RNA-seq of BAT

RNA extraction: After mouse autopsies, BAT was collected and rapidly frozen in liquid nitrogen. Total RNA was extracted from the tissue via TRIzol® Reagent according to the manufacturer’s instructions. The RNA quality was subsequently determined via a 5,300 Bioanalyzer (Agilent) and quantified via an ND-2000 (NanoDrop Technologies). Only high-quality RNA samples (OD_260/280_ = 1.8 ~ 2.2, OD_260/230_ ≥ 2.0, RQN ≥ 6.5, 28S:18S ≥ 1.0, >1 μg) were used to construct the sequencing library.

Library preparation and sequencing: RNA purification, reverse transcription, library construction and sequencing were performed at Shanghai Majorbio Biopharm Biotechnology Co., Ltd. (Shanghai, China) according to the manufacturer’s instructions. The BAT RNA-seq transcriptome library was prepared following Illumina® Stranded mRNA Prep, Ligation (San Diego, CA) using 1 μg of total RNA. Briefly, messenger RNA was first isolated via the polyA selection method with oligo (dT) beads and then fragmented with fragmentation buffer. Second, double-stranded cDNA was synthesized via a Super Script double-stranded cDNA synthesis kit (Invitrogen, CA) with random hexamer primers. The synthesized cDNA was subsequently subjected to end repair, phosphorylation and adapter addition according to the library construction protocol. Libraries were size selected for cDNA target fragments of 300 bp on 2% LowRange Ultra Agarose followed by PCR amplification via Phusion DNA polymerase (NEB) for 15 PCR cycles. After quantification with a Qubit 4.0, the sequencing library was generated on a NovaSeq X Plus platform (PE150) via a NovaSeq Reagent Kit.

Quality control and read mapping: The raw paired-end reads were trimmed and quality controlled via fastp (Chen et al., 2018) with default parameters. Then, the clean reads were separately aligned to the reference genome in orientation mode via HISAT2 (Kim et al., 2015) software. The mapped reads of each sample were assembled via StringTie (Pertea et al., 2015) via a reference-based approach.

Differential expression analysis and functional enrichment: To identify DEGs (differentially expressed genes) between two different samples, the expression level of each transcript was calculated according to the transcripts per million reads (TPM) method. RSEM (Li and Dewey, 2011) was used to quantify gene abundances. Essentially, differential expression analysis was performed via DESeq2 (Love et al., 2014) or DEGseq (Wang et al., 2010). DEGs with |log2FC| ≥ 1 and FDR < 0.05 (DESeq2) or FDR < 0.001 (DEGseq) were considered significantly differentially expressed genes. In addition, functional enrichment analysis, including GO and KEGG analyses, was performed to identify which DEGs were significantly enriched in GO terms and metabolic pathways at a Bonferroni-corrected *p* value<0.05 compared with the whole-transcriptome background. GO functional enrichment and KEGG pathway analyses were carried out via Goatools and Python SciPy software, respectively.

Alternative splicing event identification: All alternative splicing events that occurred in our sample were identified via the recently released program rMATS (Shen et al., 2014). Only the isoforms that were similar to the reference or comprised novel splice junctions were considered, and the splicing differences were detected as exon inclusion, exclusion, alternative 5′, 3′, and intron retention events.

### 2.9 RT-PCR

According to Guo et al. (2022), total RNA was extracted from tissues using Trizol reagent and reverse-transcribed into complementary DNA (cDNA) with the Maxima H-Fus First-Strand DNA Synthesis Kit (Thermo Fisher, Cat. No. K1682). The target genes were amplified using SYBR Green-based quantitative polymerase chain reaction (qPCR) Mix on an ABI Prism 7,500 qPCR System. All data were normalized to the reference gene 36b4.

Reverse transcription was performed following the manufacturer’s instructions. A 10 μL qPCR reaction system was used in this study: 1 μL of cDNA, 0.3 μL of forward primer, 0.3 μL of reverse primer, 5 μL of SYBR Premix Ex Taq™, and 3.4 μL of RNase-free H₂O. Primer sequences are listed in Supplementary Table S3. The qPCR program consisted of 40 cycles: initial denaturation at 95 °C for 30 s, followed by denaturation at 95 °C for 10 s and annealing/extension at 60 °C for 25 s.

### 2.10 16S rRNA

Total microbial genomic DNA was extracted from cecal contents using the FastPure Stool DNA Isolation Kit (MJYH, Shanghai, China). After quality and concentration assessment by 1.0% agarose gel electrophoresis and a NanoDrop2000 spectrophotometer (Thermo Scientific, USA), the DNA was stored at −80 °C for subsequent use. Targeting the V3-V4 hypervariable region of the bacterial 16S rRNA gene, PCR amplification was performed on a BIO-RAD T100 thermal cycler using the primer pair 338F (5′-ACTCCTACGGGAGGCAGCAG-3′) and 806R (5′-GGACTACHVGGGTWTCTAAT-3′). The PCR reaction system had a final volume of 20 μL, containing key reagents such as Fast Pfu polymerase and dNTPs. The cycling conditions were as follows: initial denaturation at 95 °C for 3 min, followed by 27 cycles of denaturation at 95 °C for 30 s, annealing at 55 °C for 30 s, and extension at 72 °C for 45 s, with a final extension at 72 °C for 10 min and holding at 4 °C. After recovery from 2% agarose gel, PCR products were purified using the Yuhua PCR Clean-Up Kit (Shanghai, China) and quantified with a Qubit 4.0 fluorometer (Thermo Fisher Scientific, USA). Purified amplicons were pooled in equimolar amounts and subjected to paired-end sequencing on an Illumina NextSeq2000 platform (Illumina, USA) by Majorbio Bio-Pharm Technology Co., Ltd. (Shanghai, China).

Raw sequencing data were quality-filtered with fastp and merged with FLASH, followed by denoising using the DADA2 plugin in the Qiime2 pipeline to obtain amplicon sequence variants (ASVs). Each sample was rarefied to 20,000 sequences (average Good’s coverage: 97.90%). Taxonomic annotation of ASVs was performed using the Naive Bayes classifier in Qiime2 against the SILVA 16S rRNA database (v138). Metagenomic functional prediction was conducted with PICRUSt2 following standard protocols, including core steps such as sequence alignment, phylogenetic tree construction, 16S rRNA gene copy number normalization, and pathway annotation.

### 2.11 Statistical analysis

Statistical analyses were performed via IBM SPSS Statistics 23.0. One-way ANOVA followed by Duncan’s multiple range test was used to assess differences among groups, with *p* < 0.05 considered statistically significant. Graphs were generated via GraphPad Prism 9.5.

Transcriptomic, lipidomic, and microbiome data analyses were carried out using the Majorbio Cloud platform (see Footnote 1).

## 3 Results

### 3.1 PTE promotes lipid utilization and energy expenditure to ameliorate high-fat diet-induced obesity phenotype

#### 3.1.1 Ameliorates high-fat diet-induced mouse obesity without altering energy intake

**Figure 1:**
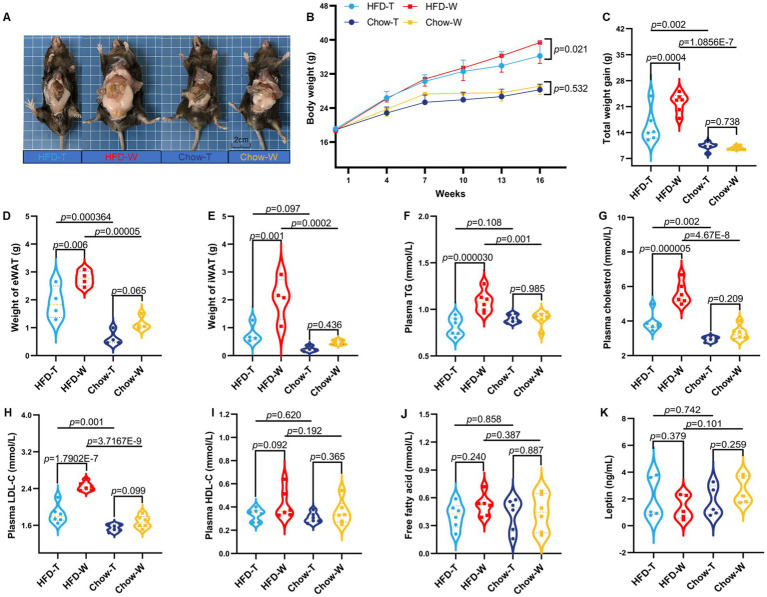
Effects of Pu-erh tea intervention on metabolic phenotypes in high-fat diet–fed mice. **(A)** Representative gross anatomy of mice (scale bar = 2 cm). **(B)** Dynamic monitoring of body weight. **(C)** Body weight gain. **(D)** Weight of epididymal white adipose tissue (eWAT). **(E)** Weight of inguinal white adipose tissue (iWAT). **(F–K)** Serum levels of triglycerides (TG), total cholesterol (TC), low-density lipoprotein cholesterol (LDL-c), high-density lipoprotein cholesterol (HDL-c), free fatty acids (FFA), and leptin, respectively. *N* = 6, data are presented as mean ± SEM. Statistical analysis was performed using one-way ANOVA followed by Tukey’s multiple comparison test.

**Figure 2:**
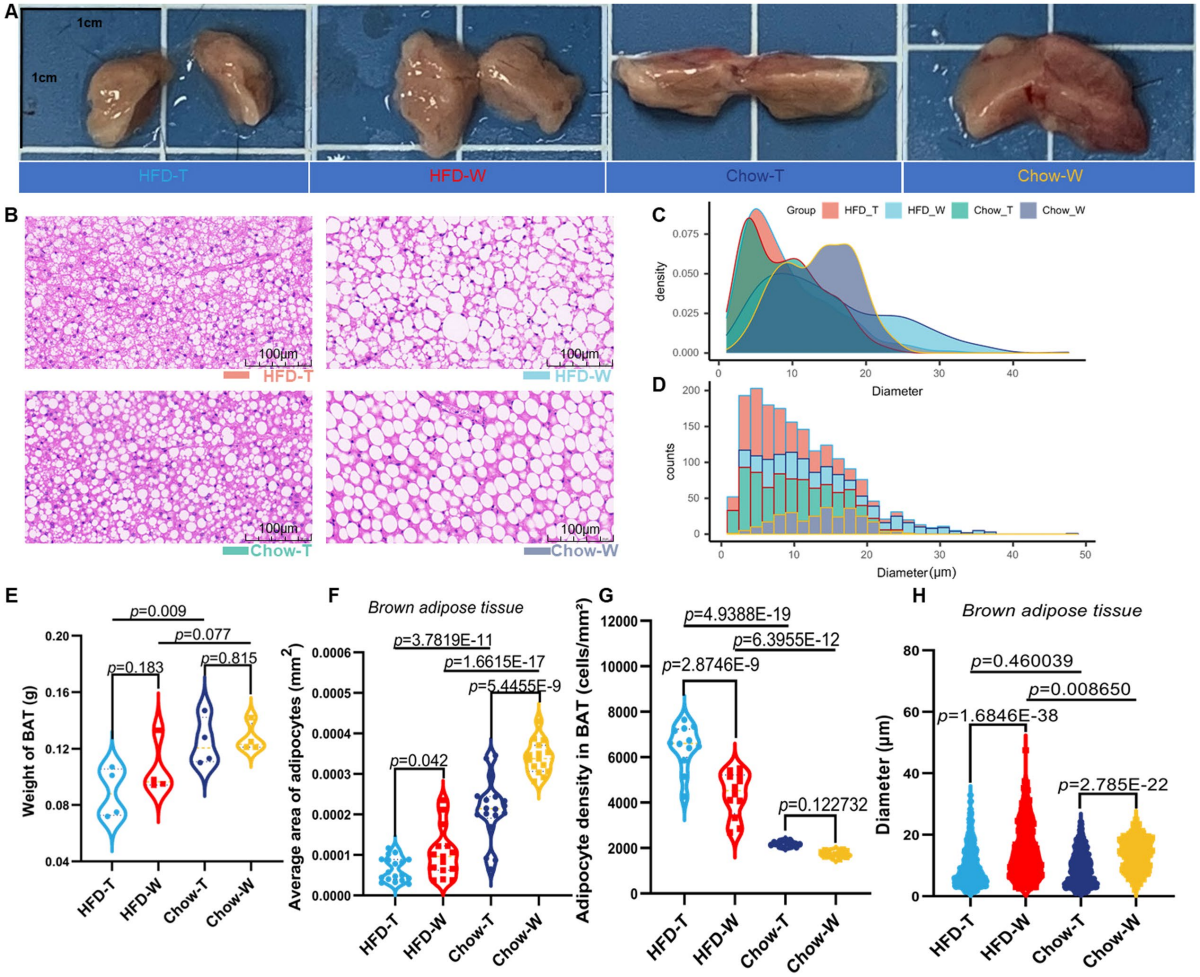
Pu-erh tea intervention promotes BAT remodeling and improves adipocyte morphology. **(A)** Representative images of BAT (scale bar = 1 cm). **(B)** Representative H&E-stained images (scale bar = 100 μm). **(C,D)** Frequency distribution of lipid droplet diameters. **(E)** Wet weight of BAT. **(F)** Quantification of average adipocyte area in BAT. **(G)** Quantification of adipocyte density in BAT. **(H)** Quantification of average lipid droplet diameter in BAT. *n* = 6 per group. Data are presented as mean ± SEM. Statistical significance was determined by one-way ANOVA followed by Tukey’s *post hoc* test.

To evaluate the effect of PTE on diet-induced obesity phenotype, we established a 16-week intervention experiment and divided mice into four groups: HFD_T, HFD_W, Chow_T and Chow_W (Supplementary Figure S1A). At week 16, mice in the HFD_W group exhibited a significantly larger body size, while those in the HFD_T group maintained a more compact body size (Figure 1A; Supplementary Figure S1B). Dynamic body weight monitoring results showed that PTE inhibited HFD-induced body weight gain (Figure 1B). Compared with the HFD_W group, the HFD_T group showed a significant reduction in body weight gain (Figure 1C), and the weights of epididymal white adipose tissue (eWAT), inguinal white adipose tissue (iWAT), and BAT were also significantly decreased (Figures 1D,E, 2E), indicating that PTE alleviated HFD-induced fat accumulation. Serum lipid analysis further revealed that PTE significantly reduced the levels of triglycerides (TG), total cholesterol (TC), and low-density lipoprotein cholesterol (LDL-c) in HFD-fed mice (Figures 1F–H). By contrast, serum HDL-c and free fatty acid levels did not differ significantly among the experimental groups (Figures 1I,J). Food intake monitoring showed that there was no significant difference in weekly food intake among the four groups (Supplementary Figure S1E). Since the HFD has a higher caloric density, HFD-fed mice had a higher cumulative energy intake (Supplementary Figure S1F). Notably, however, there was no significant difference in total cumulative energy intake between the HFD_T and HFD_W groups, indicating that PTE inhibits fat accumulation in HFD-fed mice not by reducing energy intake. Consistently, PTE did not alter serum leptin levels (Figure 1K), reaffirming that PTE exerts its effects not by enhancing satiety or reducing energy intake. Thus, we hypothesize that PTE may counteract the adverse effects of HFD by promoting energy expenditure.

#### 3.1.2 Rewires energy substrate utilization preference and promotes fatty acid oxidation in mice

**Figure 3:**
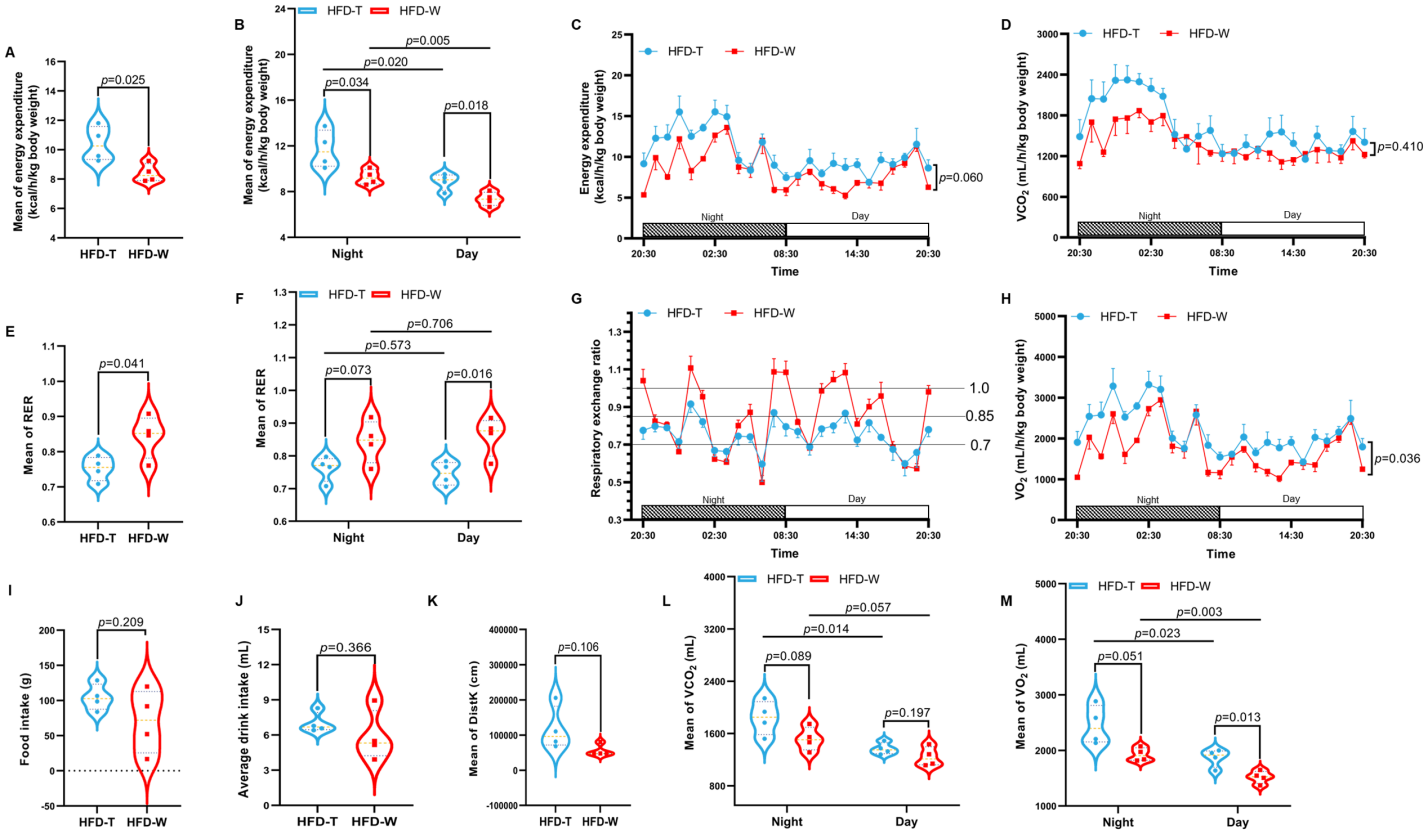
Effects of Pu-erh tea on the metabolic parameters in mice. **(A)** Average energy expenditure (EE). **(B)** Day–night differences in EE. **(C)** Dynamic monitoring curve of EE. **(D)** Dynamic monitoring curve of carbon dioxide production (VCO_2_). **(E)** Average respiratory exchange ratio (RER). **(F)** Day–night differences in RER. **(G)** Dynamic monitoring curve of RER. **(H)** Dynamic monitoring curve of oxygen consumption (VO_2_). **(I)** Food intake. **(J)** Activity level. **(K)** Water intake. **(L)** Average VCO_2_. **(M)** Average VO_2_. *n* = 4 mice per group. Data are presented as mean ± SEM. Statistical significance was determined using Student’s *t*-test. Light–dark cycles are indicated at the bottom: white bars represent the light phase (ZT0–12), and black bars indicate the dark phase (ZT12–24).

The above hypothesis was validated by metabolic cage monitoring, which showed that PTE significantly increased energy expenditure (EE) in HFD-fed mice (Figure 3A). Compared with the HFD_W group, the HFD_T group exhibited a marked elevation in heat production throughout the diurnal cycle (Figures 3B,C), indicating that PTE enhances whole-body energy expenditure regardless of whether the mice were at rest or active. Within 24 h, carbon dioxide production was significantly higher in the HFD_T group than in the HFD_W group (Figures 3D,L). Consistently, the mean oxygen consumption was also significantly elevated in the HFD_T group compared with the HFD_W group. When analyzed separately during the light and dark phases, oxygen consumption in both groups was markedly higher during the dark phase than during the light phase, in accordance with the nocturnal activity pattern of mice. Notably, although oxygen consumption in the HFD_T group was higher than that in the HFD_W group during both phases, the difference was not statistically significant during the dark phase, whereas a significant difference was observed during the light phase (Figures 3H,M). These results suggest that Pu-erh tea may predominantly enhance resting metabolic rate. To explore the mechanism underlying PTE-induced thermogenesis, we calculated the ratio of carbon dioxide production to oxygen consumption to obtain the Respiratory Exchange Ratio (RER), which is used to assess the mice’s substrate utilization preference. The results showed that the average RER of the HFD_T group was significantly lower than that of the HFD_W group (Figures 3E,F); the RER of mice in the HFD_T group was mainly between 0.7 and 0.85, while that of mice in the HFD_W group was mostly higher than 0.85 (Figure 3G), indicating enhanced fat oxidation in the HFD_T group.

In summary, PTE increases heat production by promoting fat utilization. During the monitoring period, there were no significant differences in food intake, water intake, or activity distance among the groups (Figures 3I–K), suggesting that the metabolic benefits of PTE are not dependent on the action of the central nervous system (sympathetic nerve activation) but may be mediated through peripheral tissues such as adipose tissue (Gliniak et al., 2025).

### 3.2 PTE activates BAT thermogenic function and promotes energy expenditure

Increased thermogenesis with a metabolic profile preferring fat as the energy substrate is consistent with the typical phenotype of activated thermogenic function in BAT (Mouisel et al., 2025). Considering that BAT is often in a state of functional suppression under high-fat diet conditions (Maurer et al., 2021), we hypothesize that PTE may promote systemic thermogenesis by activating BAT function.

#### 3.2.1 Improves BAT morphology and promotes lipid droplet multilocularization

Gross examination showed that BAT in the HFD_W group was enlarged and pale, while BAT in the HFD_T group was significantly reduced in volume and darker in color (Figure 2A). Histological analysis revealed that brown adipocytes in the HFD_W group were enlarged, containing large lipid droplets with obvious vacuolation, indicating a “whitening” transition of BAT. In contrast, mice in the HFD_T group retained the typical morphology of multilocular adipocytes, with smaller and densely arranged lipid droplets (Figure 2B). Quantitative data demonstrated that compared with the HFD_W group, the HFD_T group had a significant increase in the number of brown adipocytes, along with a marked decrease in the average area of adipocytes and the diameter of lipid droplets (Figures 2F–H), indicating that PTE could inhibit adipocyte hypertrophy and promote BAT remodeling. In addition, frequency distribution analysis showed that the diameter of lipid droplet vacuoles in the HFD_T group shifted toward smaller sizes with a more uniform distribution (Figures 2C,D). Since smaller brown adipocytes are usually associated with higher mitochondrial density and thermogenic potential (Peng et al., 2024), these results suggest that PTE may enhance the metabolic activity of BAT.

#### 3.2.2 Rewires the BAT lipid profile

The essence of BAT morphological changes lies in alterations in lipid molecular composition and metabolic remodeling. To further clarify the lipid molecular mechanisms by which PTE regulates BAT function, we performed untargeted lipidomic analysis on BAT based on the aforementioned morphological findings.

**Figure 4:**
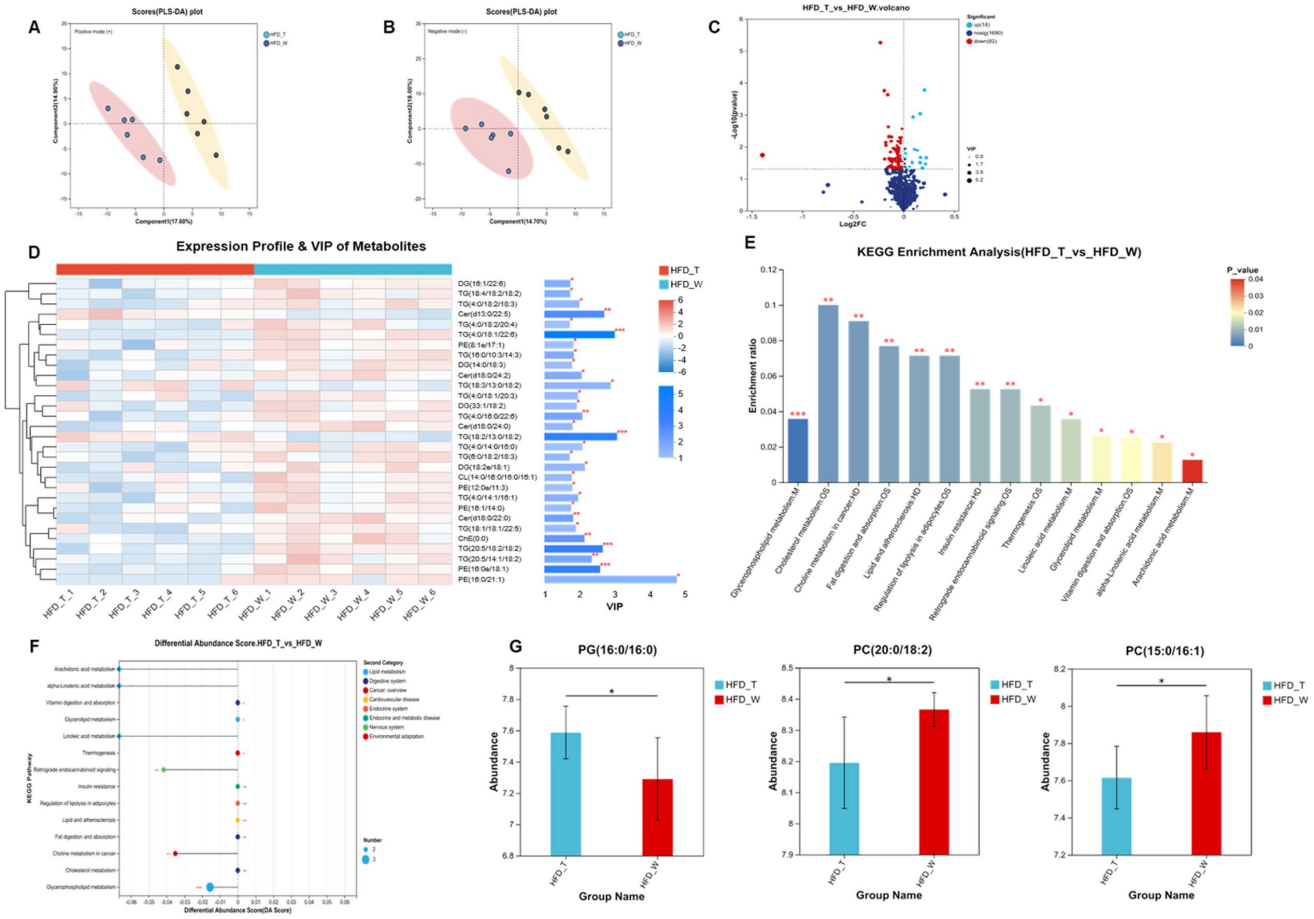
Pu-erh tea intervention remodels the lipidomic profile. **(A,B)** PLS-DA score plot of lipidomic data. **(C)** Volcano plot of differential lipids (*p* < 0.05, VIP_pred_OPLS-DA > 1). **(D)** Top 30 lipid species by VIP ranking. **(E)** KEGG pathway enrichment of differential lipid metabolites. **(F)** Differential abundance score. *n* = 6 mice per group. *p < 0.05, **p < 0.01, ***p < 0.001 indicate significant correlations.

A PLS-DA score plot was used to visually assess the classification performance of the model. The plot demonstrated a clear separation in the lipid profiles of BAT between the HFD_T and HFD_W groups, suggesting that tea consumption effectively altered the lipid composition of BAT in mice (Figures 4A,B). Untargeted lipidomics revealed 100 significantly differentially abundant lipids (DALs) between the HFD_T and HFD_W groups (Figure 4C), spanning key lipid classes, including glycerophospholipids (GP), glycerolipids (GL), sphingolipids (SP), and sterol lipids (ST) (Figure 4D). KEGG pathway enrichment analysis revealed that differentially accumulated lipids (DALs) were enriched in Glycerophospholipid metabolism, Linoleic acid metabolism, alpha-linolenic acid metabolism, Arachidonic acid metabolism, and other lipid metabolism pathways, with all these pathways exhibiting negative enrichment scores (Figures 4E,F). Further analysis of the specific variation characteristics of differential lipids in the aforementioned pathways identified a total of 3 differentially accumulated lipids: PG (16:0/16:0) showed an upward trend, while PC (20:0/18:2) and PC (15:0/16:1) exhibited a downward trend (Figure 4G). These results indicate that PTE exerts a targeted regulation on lipid metabolism, mainly affecting the metabolic networks of glycerophospholipids and polyunsaturated fatty acids.

#### 3.2.3 Activates mitochondrial energy metabolism-related pathways in BAT

**Figure 5:**
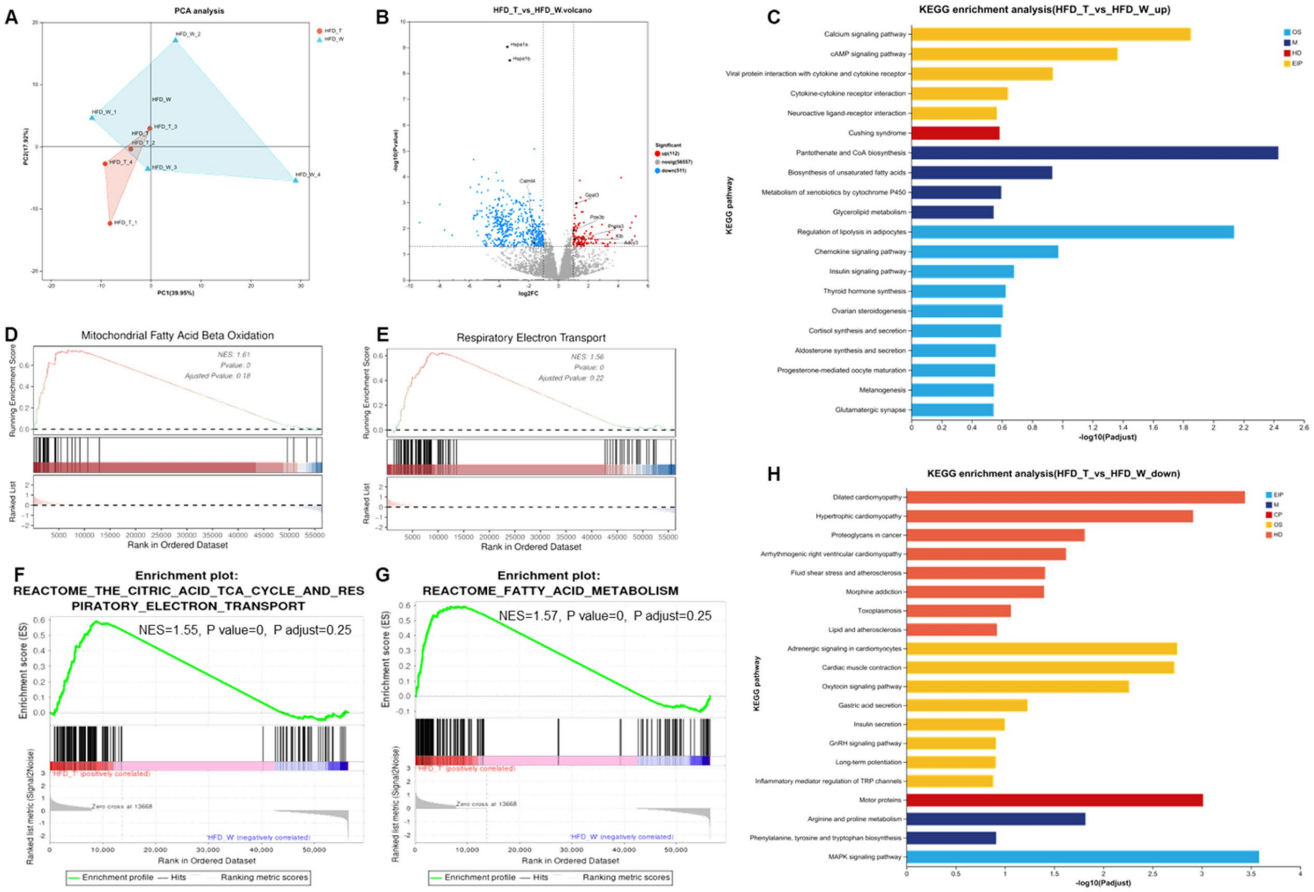
Pu-erh tea intervention remodels the transcriptomic program of brown adipose tissue and activates the lipolysis–thermogenesis axis. **(A)** Principal component analysis plot. **(B)** Volcano plot of differentially expressed genes (DEGs). **(C)** KEGG pathway enrichment of upregulated genes. **(D–G)** GSEA enrichment of DEGs. **(H)** KEGG pathway enrichment of downregulated genes. *n* = 4 mice per group. Enrichment analyses were adjusted by Benjamini–Hochberg method.

To explore the regulatory mechanism of PTE, we performed RNA-seq analysis on BAT. Principal component analysis (PCA) revealed a tendency toward clustering of gene expression profiles among the different experimental groups (Figure 5A). A total of 623 differentially expressed genes (DEGs) were identified in BAT between HFD_T and HFD_W mice (|log₂FC| ≥ 1, *p* < 0.05), including 112 upregulated and 511 downregulated genes (Figure 5B). KEGG enrichment analysis of PTE-upregulated genes showed significant enrichment in the cAMP/Calcium signaling pathway, Biosynthesis of unsaturated fatty acids, and Glycerolipid metabolism (Figure 5C). These results indicate that PTE may enhance BAT thermogenesis and fat utilization capacity by regulating BAT thermogenic signal transduction and optimizing lipid synthesis and catabolism. The downregulated differentially expressed genes were significantly enriched in pathways such as the MAPK signaling pathway, Dilated cardiomyopathy, and Hypertrophic cardiomyopathy (Figure 5H).

To clarify the regulatory role of PTE intervention in the core metabolic function of BAT under obese conditions, we performed Gene Set Enrichment Analysis (GSEA) on the DEGs. Using the screening criteria of |NES| ≥ 1.5, *p* value < 0.05, and P adjust < 0.25, two significantly positively enriched gene sets related to BAT thermogenesis and lipid metabolism were identified: mitochondrial fatty acid beta oxidation (Figure 5D) and respiratory electron transport (Figure 5E). Mitochondrial fatty acid *β*-oxidation is the core pathway through which BAT decomposes fatty acids to produce the energy substrate acetyl-CoA. Activation of this pathway indicates that PTE intervention can enhance BAT’s ability to decompose and utilize fats, providing sufficient substrates for subsequent energy conversion (thermogenesis). Mitochondrial respiratory electron transport is the key link in oxidative phosphorylation, responsible for converting energy generated from fatty acid β-oxidation into ATP or heat (thermogenesis). Activation of this pathway suggests that the energy conversion function of BAT mitochondria in the HFD_T group is enhanced, enabling more energy from fat decomposition to be released as heat. Thus, the two pathways exhibit a functional synergistic relationship between substrate supply and energy conversion: the former provides energy substrates by decomposing fats, while the latter converts substrate energy into heat through the respiratory chain. These results indicate that PTE’s regulation of BAT function is not limited to a single link but enhances BAT metabolic activity by synergistically promoting fat decomposition and energy conversion.

To verify the accuracy of the RNA-seq results, four differentially expressed genes (DEGs)—*Pde3b*, *Elovl3*, *Cyp1a1*, and *Chac1*—were randomly selected for RT-qPCR validation (Supplementary Figures S2A,B). The results showed that the expression levels of *Pde3b* and *Elovl3* in the HFD_T group were significantly higher than those in the HFD_W group, while the expression levels of *Cyp1a1* and *Chac1* were significantly lower in the HFD_T group compared with the HFD_W group. The expression trends of these genes detected by RT-qPCR were consistent with the RNA-seq data, confirming the stability and reliability of the transcriptomic sequencing results.

### 3.3 Integrated analysis reveals that the *Gpat3*-regulated glycerophospholipid metabolism pathway may be a key pathway for pte to promote systemic energy expenditure

**Figure 6:**
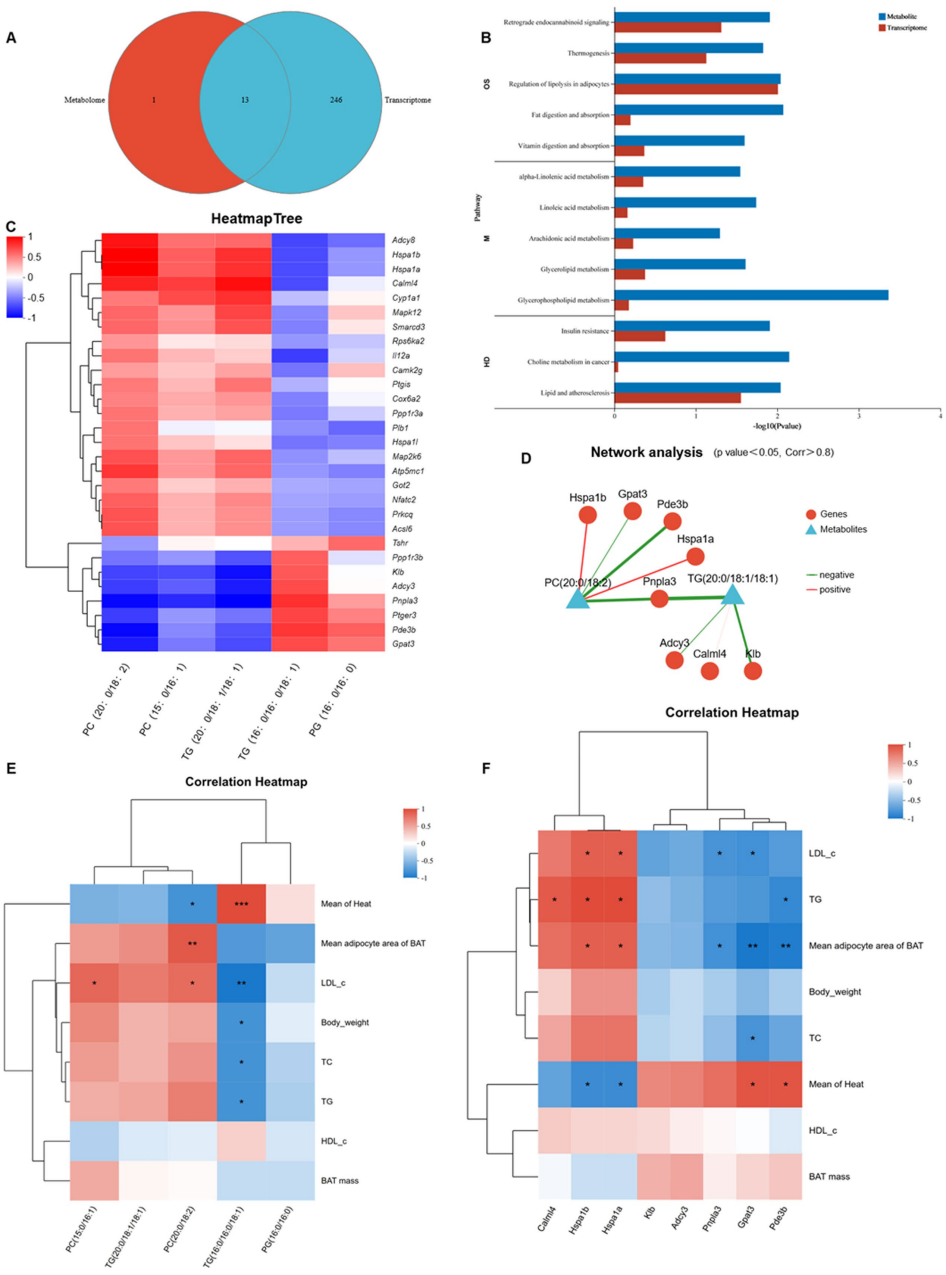
Integrated transcriptomic and lipidomic analysis reveals shared metabolic pathways. **(A)** Venn diagram of KEGG pathways enriched by DEGs and DALs. **(B)** KEGG pathway enrichment plots of DEGs and DALs. **(C)** Correlation analysis between genes and lipids. **(D)** Network analysis. **(E)** Correlation analysis between lipids and phenotypes. **(F)** Correlation analysis between genes and phenotypes.

To clarify the functional association between transcriptional regulation and lipid metabolism, common KEGG pathway enrichment analysis was performed for differential genes and differential metabolites. A total of 13 pathways were identified, including Regulation of lipolysis in adipocytes, Retrograde endocannabinoid signaling, Linoleic acid metabolism, Insulin resistance, Glycerophospholipid metabolism, and Thermogenesis, among which 5 pathways are related to lipid metabolism (Figures 6A,B). The corresponding results of genes, KEGG pathways, and lipid metabolites are presented in Supplementary Table S4, which includes 29 DEGs and 5 DALs. A correlation heatmap of genes and metabolites was constructed (Figure 6C), leading to the identification of 8 key genes and 5 key lipid molecules.

First, common enrichment analysis was performed on these genes and lipid molecules. The results showed that they were co-enriched in 5 key pathways, among which the pathways related to lipid metabolism were Glycerolipid metabolism and Glycerophospholipid metabolism (Supplementary Figure S2C). *Pnpla3*, *Gpat3*, TG (20:0/18:1/18:1), and TG (16:0/16:0/18:1) were co-enriched in Glycerolipid metabolism. *Gpat3*, PC (20:0/18:2), PC (15:0/16:1), and PG (16:0/16:0) were co-enriched in Glycerophospholipid metabolism. Spearman correlation analysis further revealed that *Pnpla3* was significantly negatively correlated with TG (20:0/18:1/18:1) (corr = −0.928, *p* = 0.000863). *Gpat3* was significantly negatively correlated with PC (20:0/18:2) (corr = −0.833, *p* = 0.0102) (Figure 6D; Supplementary Figure S2D).

Correlation analysis was performed between these 8 key genes and mouse phenotypic indicators, with the results shown in Figure 6E. *Pde3b* was significantly positively correlated with average heat production, and significantly negatively correlated with the average area of BAT adipocytes and serum TG levels. *Gpat3* was significantly positively correlated with average heat production, and significantly negatively correlated with the average area of BAT adipocytes as well as serum LDL-c and TC levels. *Hspa1a* and *Hspa1b* were significantly negatively correlated with average heat production, and significantly positively correlated with obesity-related indicators. Spearman correlation analysis between the identified 5 key DALs and phenotypic indicators revealed that PC (20:0/18:2) was significantly negatively correlated with average heat production, and significantly positively correlated with the average area of BAT adipocytes and serum LDL-c levels; TG (16:0/16:0/18:1) was significantly positively correlated with average heat production, and significantly negatively correlated with obesity-related indicators (Figure 6F).

Based on the above results and combined with the roles of genes and lipid molecules in KEGG pathways, we can speculate that under PTE intervention, *Gpat3* and PC (20:0/18:2) in BAT may be key genes and lipid molecules regulating systemic thermogenesis. *Gpat3* may reduce the content of PC (20:0/18:2) in BAT by regulating the glycerophospholipid metabolism pathway, thereby enhancing systemic thermogenesis to resist lipid accumulation.

### 3.4 Gut microbiota is a key mediator for PTE-induced thermogenesis activation and metabolic improvement

#### 3.4.1 PTE specifically regulates obesity-related gut microbial structure

Given the critical role of the gut microbiota in regulating host energy metabolism and adipose tissue function (Kuang et al., 2019; Wang Y. et al., 2023), we performed 16S rRNA sequencing on fecal samples to evaluate the contribution of gut microbial alterations to the metabolic benefits of PTE.

**Figure 7:**
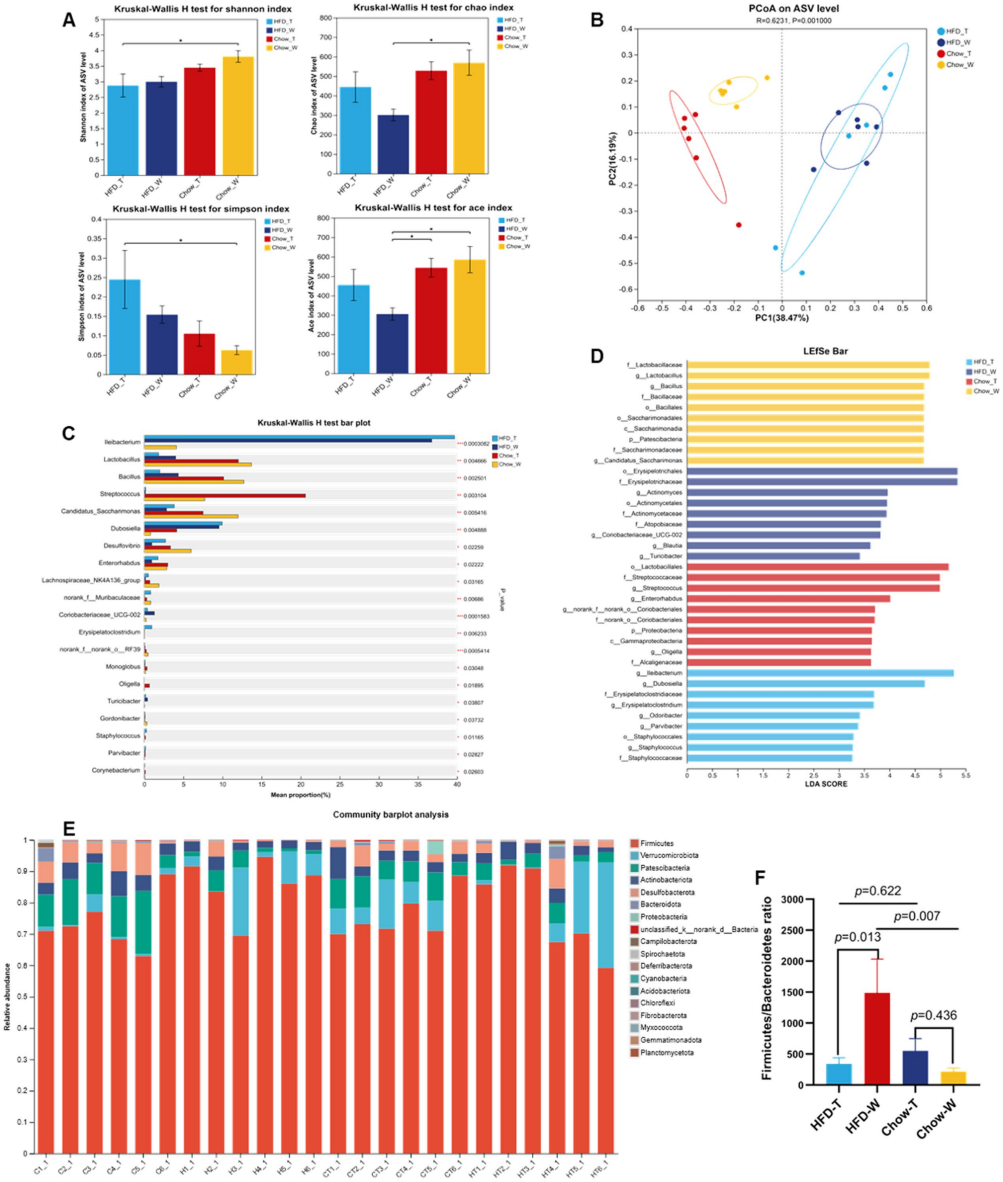
PTE intervention remodels gut microbiota composition. **(A)** Alpha diversity boxplot. **(B)** Beta diversity PCoA plot (Bray-Curtis). **(C)** Genus-level relative abundances compared by Wilcoxon rank-sum test. **(D)** LEfSe bar. **(E)** Heatmap of microbial phyla abundance. **(F)** Ration of Firmicutes to Bacteroidetes. *n* = 6 per group. Data are presented as mean ± SEM. One-way ANOVA with Tukey’s post hoc test or Wilcoxon rank-sum test was used as appropriate. *p < 0.05, **p < 0.01, ***p < 0.001.

Compared with the Chow_W group, the Chao1 and Ace indices were significantly decreased in the HFD_W group (Figure 7A), indicating a reduction in microbial richness. PTE intervention could partially restore microbial richness, consistent with previous studies (Ye et al., 2021). *β*-Diversity analysis further demonstrated distinct clustering among groups, with a separation between HFD_T and HFD_W in the PCoA plots (Figure 7B), suggesting that marked microbial community remodeling was induced by PTE. In line with earlier studies (Chen et al., 2023; Du W. et al., 2025), a HFD significantly increased the Firmicutes/Bacteroidetes (F/B) ratio, a dysbiosis marker associated with metabolic imbalance. PTE treatment reversed these alterations, restoring the F/B ratio to control levels (Figures 7E,F).

A genus-level comparison revealed that *Allobaculum*, *Blautia*, and *Coriobacteriaceae_UCG-002* were significantly elevated in HFD_W, whereas *Erysipelatoclostridium*, *norank_f__Muribaculaceae*, *Lactococcus* and *NK4A214_group*, associated with short-chain fatty acid production and energy homeostasis (Su et al., 2024; Sun et al., 2024), were enriched in HFD_T (Figures 7C,D). These findings suggest that PTE modulates the gut microbiota by promoting beneficial bacteria and suppressing potential pathobionts, thereby restoring the intestinal ecological balance.

#### 3.4.2 Prediction of gut microbial metabolic functions

Based on the eggNOG and KEGG databases, PICRUSt2 was employed in this study to predict and analyze the functional potential of the gut microbiota. The results of eggNOG functional annotation revealed that the unannotated functional genes exhibited the highest abundance, followed by genes associated with amino acid transport and metabolism, as well as those related to translation, ribosomal structure, and biogenesis (Supplementary Figure S3A). At the KEGG level 2, pathways involved in carbohydrate metabolism, amino acid metabolism, and energy metabolism were significantly enriched (Supplementary Figures S3B,C). Furthermore, combined with the KEGG level 3 data, it could be further confirmed that the functions of the microbial community were associated with metabolic pathways, biosynthesis of secondary metabolites, biosynthesis of amino acids, carbon metabolism, and pyrimidine metabolism pathways (Supplementary Figures S3D,E). Previous studies have reported a significant correlation between the functions predicted by PICRUSt2 at the KEGG level 2 and the actual metabolic characteristics of the microbial community (Zhao et al., 2021), suggesting that the functional prediction results in this study can to a certain extent reflect the metabolic potential of the microbiota in carbohydrate and amino acid utilization, thereby affecting host nutrition.

## 4 Discussion

A significant reduction in body weight without a significant change in food intake indicates that the anti-obesity effect of PTE does not stem from reduced energy intake, which is consistent with the conclusions of a recent study on Tirzepatide (Zhang et al., 2025). Data from metabolic cage monitoring showed that PTE can systematically enhance energy expenditure in mice, with the core contribution derived from increased heat production.

The Respiratory Exchange Ratio (RER) is a core quantitative indicator for evaluating the body’s preference for energy substrate utilization. When RER ≈ 0.7, it indicates that the body primarily uses fatty acid *β*-oxidation as the energy source; when RER ≈ 1.0, it suggests that the body relies on carbohydrates for energy supply (Ravussin et al., 2025; Smith et al., 2018; Wang Y. et al., 2025). The dynamic changes in RER and heat production demonstrate that PTE can shift the mice’s substrate utilization preference from carbohydrate-dominant to fat-dominant, while increasing heat production. In addition, systemic energy expenditure was significantly elevated in PTE-treated mice, whereas neither food intake nor spontaneous activity showed significant changes. This result rules out energy consumption from digestive metabolism and exercise-induced thermogenesis, suggesting that the increased energy expenditure originates from BAT-mediated non-shivering thermogenesis.

GSEA analysis of the BAT transcriptome revealed that PTE significantly activates two key pathways in BAT that are core to energy metabolism: mitochondrial fatty acid beta oxidation and respiratory electron transport. Meanwhile, the citric acid TCA cycle and respiratory electron transport (NES = 1.55, *p* value = 0, P adjust = 0.25) and fatty acid metabolism (NES = 1.57, *p* value = 0, P adjust = 0.25) also showed the same upward trend (Figures 5F,G). This indicates that PTE may enhance BAT’s thermogenic capacity by synergistically upregulating the entire metabolic cascade from fatty acid decomposition and electron transport to uncoupled thermogenesis. Consistent with the above conclusions, lipid subclass remodeling (Figure 4D) in BAT lipidomic data further supports the establishment of a metabolically active lipid microenvironment, thereby favoring energy dissipation. In addition, morphological analysis of BAT showed that the HFD_T group had smaller and more numerous lipid droplets, a phenotype consistent with the conclusions from BAT transcriptomic and lipidomic analyses.

Based on 16S rRNA sequencing analysis, we found that Pu-erh tea can regulate the richness and composition of the gut microbiota: on the one hand, it enriches beneficial bacteria such as *Muribaculaceae* (Zhao H. et al., 2025; Zhu et al., 2024), and on the other hand, it inhibits potentially harmful bacteria including *Coriobacteriaceae_UCG-002*. These microbial changes may target lipolysis and thermogenesis in adipose tissue through the signaling of microbial metabolites (Zhao H. et al., 2025; Zhu et al., 2024; May and den Hartigh, 2021). The ameliorative effect of Pu-erh tea on HFD-induced metabolic disorders is achieved through the cross-organ synergistic regulation of the “intestinal microbiota-metabolites-BAT” axis, and this mechanism has been verified in studies on tea-derived bioactive components (Li et al., 2023; Wang Y. et al., 2023; Wang et al., 2021; Yang et al., 2024). Pu-erh tea and its bioactive components (e.g., theabrownin, polyphenols) can reverse HFD-induced intestinal flora dysbiosis. HFD causes an abnormal increase in the F/B ratio, which is positively correlated with fat accumulation. Pu-erh tea intervention can restore the normal F/B ratio by inhibiting the abundance of Firmicutes and enriching Bacteroidetes (Li et al., 2025; Ning et al., 2025; Zhao et al., 2026). Pu-erh tea can also specifically enrich functional flora, including bacteria with 7α-dehydroxylation activity (*Clostridium scindens, Parabacteroides distasonis*) and probiotics (*Akkermansia muciniphila*, *Lactobacillus*, Bacteroides, Bifidobacterium) (Wang et al., 2021; Yang et al., 2024; Kuang et al., 2020; Wang Y. et al., 2023; Zhao S. et al., 2025). Among these, *Akkermansia muciniphila* shows a strong negative correlation with weight gain and serves as a key core strain mediating adipose thermogenesis (Wang Y. et al., 2023).

The intestinal microbiota remodeled by Pu-erh tea establishes signaling pathways between the intestine and BAT through the production of specific metabolites. In addition to the classical short-chain fatty acids (SCFAs) and bile acids (BAs), these metabolites also include novel regulatory molecules such as succinate (Li et al., 2023). Pu-erh tea intervention can significantly increase the levels of acetate, propionate, and butyrate in the intestine and serum (Wang et al., 2021; Yang et al., 2024; Zhao S. et al., 2025). These metabolites activate GPR41/GPR43 receptors in adipose tissue, regulate the AMPK-PGC1α-UCP1 signaling pathway, directly promote fatty acid *β*-oxidation and non-shivering thermogenesis in BAT, and simultaneously induce browning of white adipose tissue to form beige fat (Wang et al., 2021; Yang et al., 2024; Zhao S. et al., 2025; Du Y. et al., 2025). Studies on summer-autumn tea aqueous extract have confirmed that its thermogenic effect is highly correlated with SCFA production and microbiota reconstruction (Yang et al., 2024). Moreover, SCFAs produced by the gut microbiota can further enhance BAT activity by activating the AMPK/SIRT1/PGC-1α pathway (Du Y. et al., 2025). Bacteria with 7α-dehydroxylation activity, such as *C. scindens*, can shift bile acid biosynthesis from the classical pathway to the alternative pathway, which is specifically manifested by increased levels of non-12α-hydroxylated BAs (non-12OH-BAs, e.g., UDCA, LCA) and decreased levels of 12α-hydroxylated BAs (12OH-BAs, e.g., CA, DCA, TCA) (Hu et al., 2025; Kuang et al., 2020; Zhao C. et al., 2025). This change activates the TGR5-DIO2-T3 axis in BAT and upregulates the expression of core genes related to fatty acid β-oxidation, such as UCP1, PGC1α, and Acox1 (Kuang et al., 2020; Zhao C. et al., 2025). Ziyang selenium-enriched green tea polysaccharide can increase the content of succinate in the colon through microbiota regulation. This metabolite is directly associated with adipocyte thermogenesis, thereby upregulating the expression of thermogenic marker proteins such as UCP1 and PGC-1α in BAT and inguinal white adipose tissue (Li et al., 2023).

The aforementioned changes in microbiota and metabolites ultimately lead to the activation of BAT function. The activation of the AMPK pathway mediated by SCFAs can accelerate the rate of mitochondrial fatty acid *β*-oxidation in BAT, and Theabrownin extracted from Fuzhuan brick tea can also promote thermogenesis through this pathway (Wang et al., 2021). The TGR5 signaling pathway triggered by bile acids can enhance the thermogenic capacity of BAT while reducing the inflammatory levels in the intestine and adipose tissue (Hu et al., 2025; Wang et al., 2021; Kuang et al., 2020). Notably, the tea-derived probiotic Eurotium cristatum exerts adipothermogenic effects by enriching *Akkermansia muciniphila*. Supplementation with *Akkermansia muciniphila* alone can replicate the effects of inhibiting fat accumulation and enhancing BAT activity (Wang Y. et al., 2023). Further verification by antibiotic experiments confirms that the metabolic ameliorative effect of Pu-erh tea is completely dependent on the intestinal microbiota, as antibiotic treatment eliminates all its efficacy (Zhao S. et al., 2025); fecal microbiota transplantation experiments directly verify the causal regulatory role of the microbiota (Du Y. et al., 2025).

Taken together, our study has clarified the mechanism by which PTE exerts anti-obesity effects through activating BAT function and remodeling the intestinal flora. Its efficacy has also been verified from multiple dimensions including systemic metabolism, organ function, molecular pathways, and intestinal microecology. However, future research can be further expanded and improved in the following aspects. Firstly, isolate and purify the main bioactive components from PTE, and verify the regulatory effects of each component on BAT thermogenic marker proteins and fatty acid β-oxidation related genes using *in vitro* BAT cell models. Further *in vivo* animal experiments should be conducted to clarify the impacts of core bioactive components on BAT function, energy metabolism, and obesity phenotypes. Secondly, establish germ-free mouse models and perform fecal microbiota transplantation experiments. Transplant the intestinal flora from PTE-treated mice into germ-free mice, and observe changes in BAT thermogenic activity, fatty acid metabolism, and obesity-related phenotypes in recipient mice to directly confirm the mediating role of the flora. Meanwhile, combine antibiotic treatment experiments to verify whether the regulation of PTE on BAT function depends on the intestinal microecology by eliminating the intestinal flora. Isolate and enrich the key functional strains remodeled by PTE, and conduct in vivo colonization experiments with these strains either individually or in combination to clarify the regulatory effects of specific strains on BAT function.

## Data Availability

The datasets presented in this study have been deposited in the Gene Expression Omnibus (GEO) database (accession number: GSE312855).

## Footnotes

1
https://cloud.majorbio.com

2
https://docs.scipy.org/doc/scipy/
