# Supplementary material for: Pu-erh tea attenuates obesity by remodeling gut microbiota and activating energy expenditure
Source: Front Microbiol. 2026 Jan 15;16:1752456. doi: 10.3389/fmicb.2025.1752456 (PMC12853662; doi:10.3389/fmicb.2025.1752456)
Supplement: Supplementary file 1 [file Data_Sheet_1.PDF]

Appendix A. Supplementary data

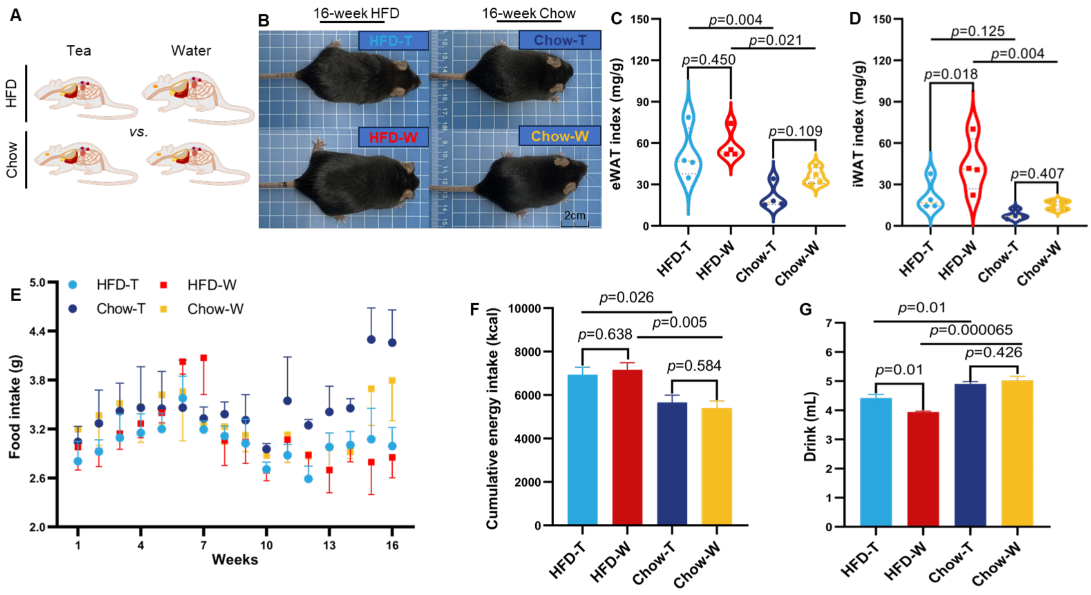

Fig S1. Experimental design and basic phenotypic characteristics of mice

(A) Experimental grouping.

(B) Representative images of mice at week 16 (scale bar = 2 cm).

(C) Epididymal white adipose tissue (eWAT) weight index.

(D) Inguinal white adipose tissue (iWAT) weight index.

(E) Food intake.

(F) Energy intake.

(G) Water intake.

Data are presented as mean ± SEM. Statistical significance was determined by one-way ANOVA followed by Tukey’s post hoc multiple comparisons test.

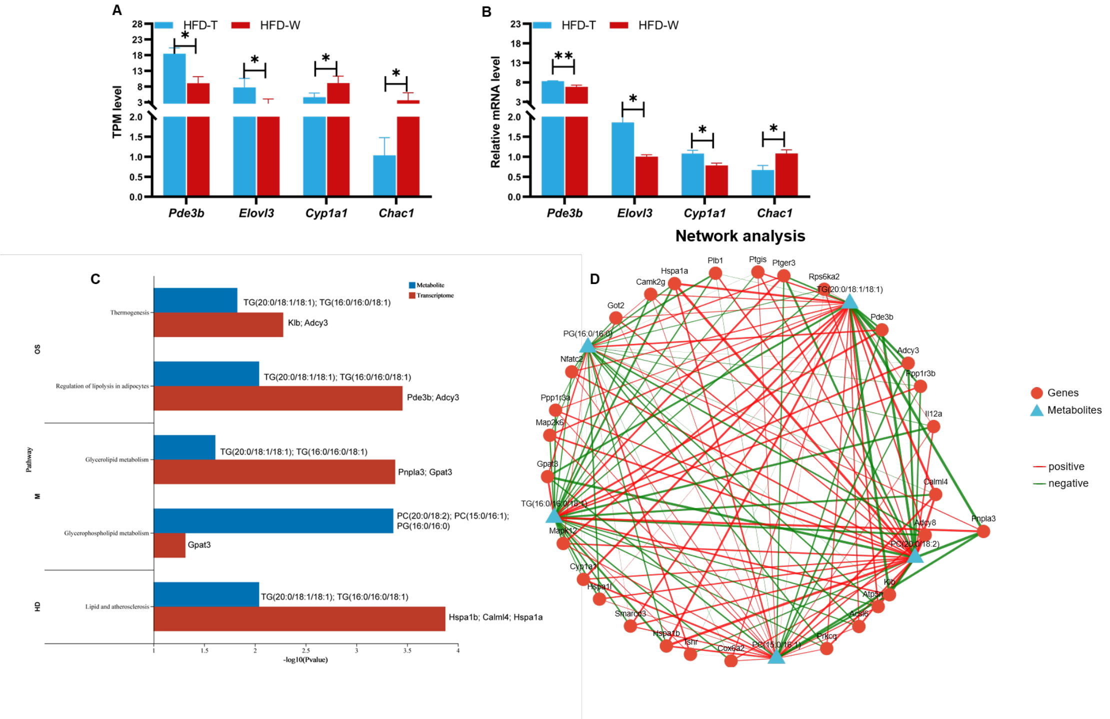

Fig. S2. Validation of gene expression and multi-omics correlation analysis

(A) Gene expression levels (TPM).

(B) Relative mRNA levels.

(C) Visualization of genes, KEGG pathways, and metabolites.

(D) Network analysis of genes and lipid molecules.

Data are presented as mean ± SEM. Statistical significance was determined by one-way ANOVA followed by Tukey’s post hoc multiple comparisons test.

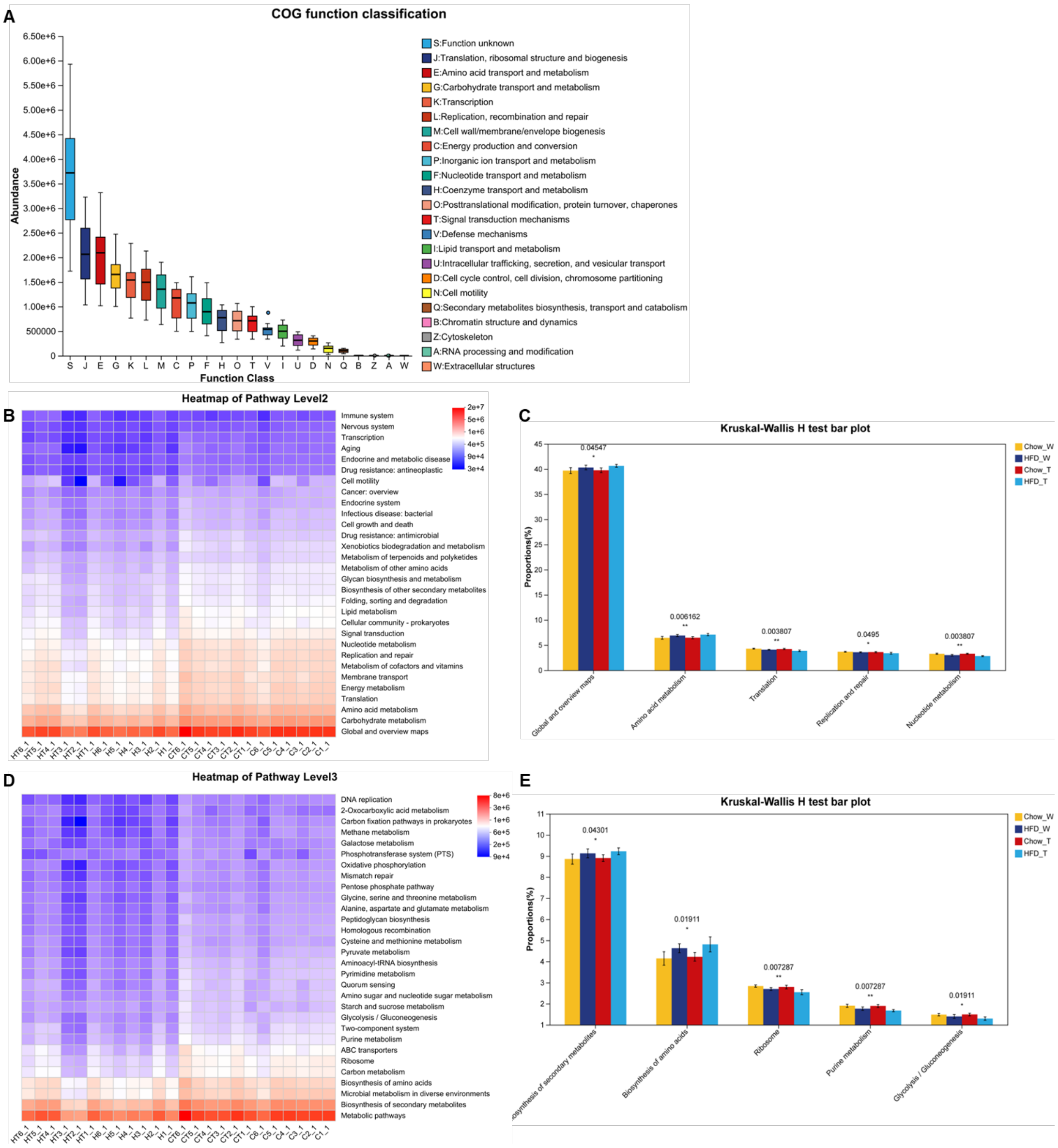

Fig. S3. Validation of gene expression and multi-omics correlation analysis

(A) COG functional classification predicted by PICRUSt2.

(B, C) Heatmaps of KEGG pathway enrichment at levels 2.

(D, E) Heatmaps of KEGG pathway enrichment at levels 3.

Table S1. Phytochemical profiles of Pu-erh tea extracts

Components	Contents（mg/g）
Gallic Acid (GA)	33.52±0.28
Gallocatechin (GC)	5.89±0.04
Epigallocatechin (EGC)	2.17±0.06
Catechin (C)	2.23±0.18
Caffeine (CAF)	112.95±0.19
Epicatechin (EC)	13.49±0.43
Epigallocatechin Gallate (EGCG)	2.40±0.20
Gallocatechin Gallate (GCG)	1.36±0.01
Epicatechin Gallate (ECG)	37.32±0.06
Total polyphenols	365.69±3.32
Tea polysaccharide	46.67±1.67

Values are mean ± SD (n = 3).

Table S2. Macronutrient distribution in normal chow and high-fat diet groups.

Parameter	HFD Diet			Chow Diet		
	Mass ratio, %	Kcal/kg	Energy supply ratio, %	Mass ratio, %	Kcal/kg	Energy supply ratio, %
Protein	23.25	930	18.14	18.8	752	20.54
Total fat	34.55	3110	60.65	5.2	468	12.79
Carbohydrate	27.20	1088	21.22	61.0	2440	66.67
	Total Kcal	5128	100.00	*Total Kcal*	3660	100.00

Table S3. The primers for RT-PCR (5′to 3′).

Gene	Primer	
36b4	Forward Primer	GAAACTGCTGCCTCACATCCG
	Reverse Primer	GCTGGCACAGTGACCTCACACG
Pde3b	Forward Primer	CCAATTCCTGGCTTACCTCA
	Reverse Primer	GTGATCGTAATCGTGCATGG
Elovl3	Forward Primer	AGCAAGGTTGTTGAACTGGGA
	Reverse Primer	GACGCTTACGCAGGATGATGA
Cyp1a1	Forward Primer	GTTTATGACACTGCATGTGG
	Reverse Primer	GAGAAACATGGACATGCAAG
Chac1	Forward Primer	GGCTTCGTTCGTGGCTATAGC
	Reverse Primer	CAGCCCTCACGGTCTTCAAG

Table S4. KEGG co-enrichment results of genes and lipid metabolites.

Pathway Description	Metabolite list	Gene list
Insulin resistance	TG(20:0/18:1/18:1) ; TG(16:0/16:0/18:1)	*Ppp1r3b; Prkcq; Rps6ka2; Ppp1r3a*
Lipid and atherosclerosis	TG(20:0/18:1/18:1) ; TG(16:0/16:0/18:1)	*Map2k6; Mapk12; Hspa1b; Calml4; Nfatc2; Il12a; Hspa1l; Camk2g; Cyp1a1; Hspa1a*
Thermogenesis	TG(20:0/18:1/18:1) ; TG(16:0/16:0/18:1)	*Adcy8; Smarcd3; Atp5mc1; Klb; Cox6a2; Adcy3; Acsl6; Rps6ka2; Mapk12*
Regulation of lipolysis in adipocytes	TG(20:0/18:1/18:1) ; TG(16:0/16:0/18:1)	*Adcy8; Pde3b; Tshr; Adcy3; Ptger3*
Fat digestion and absorption	TG(20:0/18:1/18:1) ; TG(16:0/16:0/18:1)	*Got2*
Vitamin digestion and absorption	TG(20:0/18:1/18:1) ; TG(16:0/16:0/18:1)	*Plb1*
alpha-Linolenic acid metabolism	PC(15:0/16:1) ; PC(20:0/18:2)	*Plb1*
Linoleic acid metabolism	PC(15:0/16:1) ; PC(20:0/18:2)	*Plb1*
Arachidonic acid metabolism	PC(15:0/16:1) ; PC(20:0/18:2)	*Ptgis; Plb1*
Glycerolipid metabolism	TG(20:0/18:1/18:1) ; TG(16:0/16:0/18:1)	*Pnpla3; Gpat3*
